# Supplementary material for: Multi‐dataset identification of innovative feature genes and molecular mechanisms in keratoconus
Source: J Cell Mol Med. 2024 Sep 19;28(18):e70079. doi: 10.1111/jcmm.70079 (PMC11412914; doi:10.1111/jcmm.70079)
Supplement: Supplementary file 2 — Material S2. [file JCMM-28-e70079-s002.zip › 7.GSEA analysis/4.MYRF/KEGG_TASTE_TRANSDUCTION.html]

Details for gene set KEGG\_TASTE\_TRANSDUCTION[GSEA]

|  || Dataset | MYRF.group.cls#h\_versus\_l |
| Phenotype | group.cls#h\_versus\_l |
| Upregulated in class | h |
| GeneSet | KEGG\_TASTE\_TRANSDUCTION |
| Enrichment Score (ES) | 0.38799512 |
| Normalized Enrichment Score (NES) | 1.1197989 |
| Nominal p-value | 0.28486055 |
| FDR q-value | 1.0 |
| FWER p-Value | 0.997 |
Table: GSEA Results Summary

  

Fig 1: Enrichment plot: KEGG\_TASTE\_TRANSDUCTION      
 Profile of the Running ES Score & Positions of GeneSet Members on the Rank Ordered List

  

| SYMBOL | TITLE | RANK IN GENE LIST | RANK METRIC SCORE | RUNNING ES | CORE ENRICHMENT || 1 | TAS2R10 | na | 118 | 0.801 | 0.0594 | Yes |
| 2 | TAS2R13 | na | 242 | 0.685 | 0.1096 | Yes |
| 3 | TAS2R14 | na | 334 | 0.636 | 0.1569 | Yes |
| 4 | TAS2R43 | na | 348 | 0.629 | 0.2059 | Yes |
| 5 | TAS2R46 | na | 808 | 0.495 | 0.2315 | Yes |
| 6 | TAS2R38 | na | 977 | 0.464 | 0.2631 | Yes |
| 7 | TAS2R50 | na | 1085 | 0.447 | 0.2951 | Yes |
| 8 | TAS2R31 | na | 1746 | 0.379 | 0.3058 | Yes |
| 9 | TAS2R19 | na | 1883 | 0.366 | 0.3306 | Yes |
| 10 | TAS2R20 | na | 2640 | 0.315 | 0.3335 | Yes |
| 11 | TAS2R45 | na | 2767 | 0.307 | 0.3540 | Yes |
| 12 | TAS2R42 | na | 2853 | 0.303 | 0.3753 | Yes |
| 13 | TAS2R4 | na | 3193 | 0.286 | 0.3880 | Yes |
| 14 | GRM4 | na | 4143 | 0.250 | 0.3802 | No |
| 15 | TAS2R1 | na | 4883 | 0.226 | 0.3766 | No |
| 16 | TAS2R60 | na | 6893 | 0.167 | 0.3318 | No |
| 17 | TRPM5 | na | 7416 | 0.153 | 0.3287 | No |
| 18 | TAS2R8 | na | 8028 | 0.138 | 0.3219 | No |
| 19 | CACNA1B | na | 8324 | 0.131 | 0.3237 | No |
| 20 | TAS1R3 | na | 8987 | 0.116 | 0.3137 | No |
| 21 | SCNN1A | na | 9378 | 0.108 | 0.3110 | No |
| 22 | PLCB2 | na | 9762 | 0.100 | 0.3078 | No |
| 23 | ADCY4 | na | 9919 | 0.097 | 0.3109 | No |
| 24 | PRKX | na | 11663 | 0.068 | 0.2660 | No |
| 25 | TAS2R3 | na | 12951 | 0.051 | 0.2329 | No |
| 26 | ADCY6 | na | 13405 | 0.045 | 0.2233 | No |
| 27 | GNG13 | na | 14122 | 0.036 | 0.2055 | No |
| 28 | ITPR3 | na | 17996 | -0.000 | 0.0939 | No |
| 29 | GNG3 | na | 18263 | -0.003 | 0.0865 | No |
| 30 | TAS2R9 | na | 18647 | -0.007 | 0.0760 | No |
| 31 | PRKACA | na | 19584 | -0.017 | 0.0503 | No |
| 32 | ADCY8 | na | 20359 | -0.026 | 0.0300 | No |
| 33 | CACNA1A | na | 21608 | -0.041 | -0.0028 | No |
| 34 | GNAS | na | 22466 | -0.052 | -0.0234 | No |
| 35 | TAS2R40 | na | 23260 | -0.064 | -0.0413 | No |
| 36 | TAS1R2 | na | 23777 | -0.072 | -0.0505 | No |
| 37 | TAS1R1 | na | 23941 | -0.075 | -0.0494 | No |
| 38 | GNB1 | na | 23971 | -0.075 | -0.0443 | No |
| 39 | GNAT3 | na | 24206 | -0.079 | -0.0449 | No |
| 40 | TAS2R39 | na | 27112 | -0.138 | -0.1178 | No |
| 41 | GNB3 | na | 27836 | -0.156 | -0.1264 | No |
| 42 | TAS2R5 | na | 28451 | -0.174 | -0.1305 | No |
| 43 | TAS2R41 | na | 28613 | -0.178 | -0.1212 | No |
| 44 | TAS2R16 | na | 29233 | -0.198 | -0.1235 | No |
| 45 | PRKACG | na | 31385 | -0.285 | -0.1631 | No |
| 46 | SCNN1B | na | 32203 | -0.330 | -0.1608 | No |
| 47 | KCNB1 | na | 32273 | -0.335 | -0.1366 | No |
| 48 | ASIC2 | na | 32362 | -0.340 | -0.1124 | No |
| 49 | TAS2R7 | na | 33194 | -0.406 | -0.1045 | No |
| 50 | PRKACB | na | 33438 | -0.435 | -0.0774 | No |
| 51 | SCNN1G | na | 34173 | -0.565 | -0.0543 | No |
| 52 | PDE1A | na | 34685 | -0.896 | 0.0012 | No |
Table: GSEA details [plain text format]

  

Fig 2: KEGG\_TASTE\_TRANSDUCTION      
 Blue-Pink O' Gram in the Space of the Analyzed GeneSet

  

Fig 3: KEGG\_TASTE\_TRANSDUCTION: Random ES distribution      
 Gene set null distribution of ES for **KEGG\_TASTE\_TRANSDUCTION**
